# Comparative metagenomics of three *Dehalococcoides*-containing enrichment cultures: the role of the non-dechlorinating community

**DOI:** 10.1186/1471-2164-13-327

**Published:** 2012-07-23

**Authors:** Laura A Hug, Robert G Beiko, Annette R Rowe, Ruth E Richardson, Elizabeth A Edwards

**Affiliations:** 1 Department of Cell and Systems Biology, University of Toronto, Toronto, Canada; 2 Faculty of Computer Science, Dalhousie University, Halifax, NS, Canada; 3 Civil & Environmental Engineering, Cornell University, Ithaca, NY, USA; 4 Department of Chemical Engineering and Applied Chemistry, University of Toronto, Toronto, ON, Canada

## Abstract

**Background:**

The *Dehalococcoides* are strictly anaerobic bacteria that gain metabolic energy via the oxidation of H_2_ coupled to the reduction of halogenated organic compounds. *Dehalococcoides* spp. grow best in mixed microbial consortia, relying on non-dechlorinating members to provide essential nutrients and maintain anaerobic conditions.

A metagenome sequence was generated for the dechlorinating mixed microbial consortium KB-1. A comparative metagenomic study utilizing two additional metagenome sequences for *Dehalococcoides*-containing dechlorinating microbial consortia was undertaken to identify common features that are provided by the non-dechlorinating community and are potentially essential to *Dehalococcoides* growth.

**Results:**

The KB-1 metagenome contained eighteen novel homologs to reductive dehalogenase genes. The metagenomes obtained from the three consortia were automatically annotated using the MG-RAST server, from which statistically significant differences in community composition and metabolic profiles were determined. Examination of specific metabolic pathways, including corrinoid synthesis, methionine synthesis, oxygen scavenging, and electron-donor metabolism identified the Firmicutes, methanogenic Archaea, and the ∂-Proteobacteria as key organisms encoding these pathways, and thus potentially producing metabolites required for *Dehalococcoides* growth.

**Conclusions:**

Comparative metagenomics of the three *Dehalococcoides*-containing consortia identified that similarities across the three consortia are more apparent at the functional level than at the taxonomic level, indicating the non-dechlorinating organisms’ identities can vary provided they fill the same niche within a consortium. Functional redundancy was identified in each metabolic pathway of interest, with key processes encoded by multiple taxonomic groups. This redundancy likely contributes to the robust growth and dechlorination rates in dechlorinating enrichment cultures.

## Background

The *Dehalococcoides* (*Dhc*) comprise a genus-level group of bacteria within the phylum *Chloroflexi*, notable for their ability to respire halogenated compounds including recalcitrant groundwater contaminants [1-4]. Their obligate use of halogenated organic compounds as an energy source has allowed successful development of *Dhc*-containing enrichment cultures for bioaugmentation of chlorinated ethene-contaminated sites [5,6]. Within the *Dhc* group, several strains have been isolated, and there are currently five published genome sequences available [2,7,8]. The *Dhc* genome sequences reveal small, approximately 1.4 Mb chromosomes which encode ~1500 genes and a relatively reduced metabolism [2,7,8]. Each *Dhc* strain contains a unique complement of genes that are homologs of known reductive dehalogenase genes (*rdh*s), the genes required for respiration of halogenated organic compounds [8]. The characterized reductive dehalogenases are iron-sulfur cluster- and cobalamin-containing membrane-bound components of the *Dhc* electron transport chain, catalyzing the H_2_-dependent dehalogenation of a chlorinated substrate with the resultant production of H^+^ and Cl^-^ ions [9-11]. In a peculiar example of genome streamlining, the *Dhc* genomes encode only a partial corrinoid synthesis pathway [2,12], despite a cobalamin cofactor being required for reductive dehalogenase activity and hence *Dhc* energy production and growth. Isolate cultures of *Dhc* require exogenous cobalamin amended to the media to allow growth and dechlorination [2,13-15]. A constraint-based metabolic flux model examining the *Dhc* core metabolism highlighted the streamlined nature of the *Dhc* genomes; many typical bacterial pathways, including the TCA cycle and glycolysis are incompletely encoded by this group [12].

The majority of *Dhc*-containing cultures are maintained as mixed microbial consortia, primarily because *Dhc* are notoriously difficult to isolate, but also because mixed cultures exhibit higher *Dhc* growth rates, higher *Dhc* titer, and better stability [15-17]. Additionally, mixed cultures are more robust to oxygen exposure, which can kill a pure *Dhc* culture [18]. The non-dechlorinating members of these cultures are typically fermentative and acetogenic bacteria and methanogens [5,19-21]. Organic electron donors are fermented to hydrogen and acetate, which *Dhc* can subsequently utilize. Methanogenic Archaea in the cultures occupy the same niche as dechlorinators, relying on hydrogen and acetate from fermentative and acetogenic bacteria. Collectively, the non-dechlorinating populations help to maintain a reducing environment, and synthesize vitamins and other metabolites that *Dhc* requires [22-24]. It is hypothesized that the *Dhc* can scavenge the majority of its required metabolites from the organisms extant in the mixed cultures. This hypothesis was lent weight by experiments in which *Dhc* pure cultures amended with vitamin B12 (cobalamin) or a B12-producing acetogen showed increased growth and dechlorination rates compared to *Dhc* grown without an external source of cobalamin [16]. Still, the individual roles of community members in dechlorinating consortia are not well defined. The non-dechlorinating communities coexisting with *Dhc* show distinct taxonomic compositions in different enrichments; the structure of the community might depend on the electron donor and medium conditions that the cultures are maintained on [21,25].

The KB-1 consortium is a well-defined enrichment culture originally derived from a TCE-contaminated site in southern Ontario [26]. KB-1 robustly dechlorinates PCE through trichloroethene (TCE), *cis*-dichloroethene (*cis*DCE), and vinyl chloride (VC) to ethene, and has been commercialized for use as a bioremediation tool. Previous work with KB-1 has identified the major bacterial and archaeal community members [25], as well as gene sequences for 14 unique genes homologous to reductive dehalogenases [27]. It contains multiple strains of uncharacterized *Dhc* and a single *Geobacter* sp. as active dechlorinating organisms, as well as a variety of methanogens and acetogens [25].

**Figure 1:**
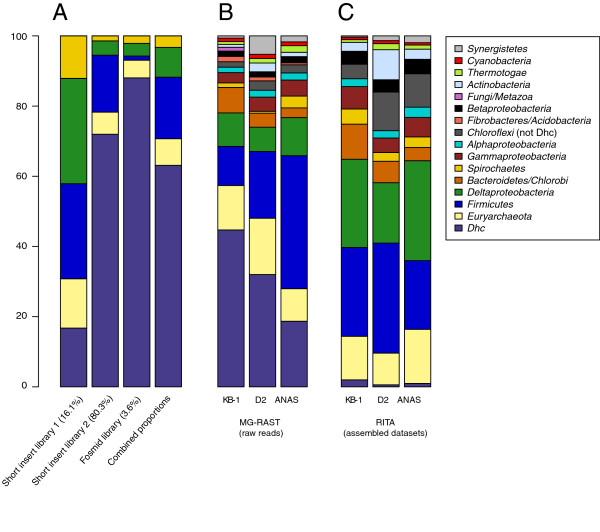
**Estimates of community composition for the three microbial consortia based on three different metrics. A**: qPCR-based estimates of the KB-1 community from the gDNA used for generation of the three sequencing libraries, and the combined expected proportions given the amount of sequence generated from each library. qPCR estimates were based on tracking 10 dominant OTUs within the consortium [30]. **B**: MG-RAST-annotated prokaryotic proportions for the metagenomes’ raw reads. The MG-RAST automated annotation used was the SEED’s phylogenetic profile. All reads from the metagenomes were used to generate the initial profiles, with hits filtered by a maximum e-value of 10^-5^ and a minimum alignment length of 100. **C**: Proportion of taxonomic assignments for the assembled metagenome datasets based on RITA taxonomic assignments. All taxonomic groups present above 1% in any of the three metagenomes were included in the proportional representations in B and C.

**Figure 2:**
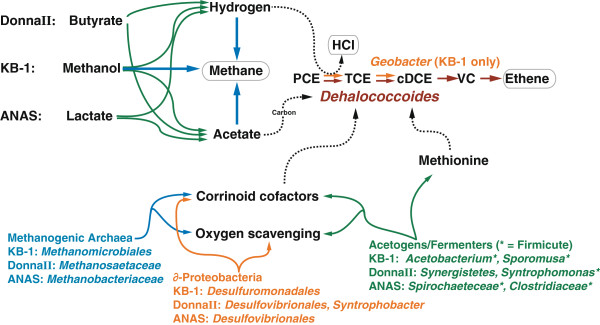
**Schematic overview of the examined metabolic processes taking place within the dechlorinating enrichment consortia, with the organisms implicated in each process highlighted.** End products are boxed in light grey. Organisms are coloured by taxonomic affiliation and process: Methanogenesis and synthesis pathways encoded by methanogenic Archaea are in blue, acetogenesis and other processes largely attributed to Firmicutes are in green, and pathways encoded by ∂-Proteobacteria are in orange. NB: A hydrogen molecule is required for each dechlorination step from PCE to ethene: only the first dechlorination reaction is depicted here.

A metagenome sequencing project for the KB-1 consortium was completed by the Joint Genome Institute (JGI), generating 103 Mb of sequence. Recently, the JGI has also sequenced metagenomes from two other *Dehalococcoides*-containing enrichment cultures: DonnaII and ANAS. The three microbial consortia, KB-1, DonnaII, and ANAS, are maintained in Toronto, Ontario, Canada, Ithaca, NY, and Berkeley, CA, respectively. They contain different strains of *Dhc*, with unique complements of *rdh* genes [7,27,28]. Prior to metagenome sequencing, a genome sequence for the *Dhc* strain in the DonnaII consortium was generated [7], and a draft genome sequence for the *Dhc* strain in the KB-1 culture has subsequently been generated from the metagenome sequence (in-house data). The consortia have each been maintained for over a decade, in different volumes, with differing growth conditions, and with different electron donors (Table 1). The KB-1 consortium is amended methanol as an electron donor, while DonnaII is amended butyrate and ANAS utilizes lactate as an electron donor. The consortia each contain methanogenic Archaea as well as acetogenic and/or fermentative organisms, but the dominant Archaea and acetogens differ taxonomically in each culture (see Figures 1, 2). In general, reference genomes for these organisms are not available from public databases. Two exceptions are the genome sequence of the δ-Proteobacterium *Geobacter lovleyi* strain SZ, which is a close relative to the *Geobacter* strain in the KB-1 consortium, and the euryarchaeote *Methanospirillum hungatei* in the DonnaII culture. Despite the physical differences in maintenance conditions and the taxonomic variation between the consortia, all three consortia are capable of the complete dechlorination of tetra- or trichloroethene to non-toxic ethene, indicating that the environmental differences present do not preclude sustained *Dhc* growth.

**Table 1:** **Physical maintenance conditions for the three *Dehalococcoides*-containing enrichment cultures**

	**KB-1**	**DonnaII**	**ANAS**
**Cultures**			
Liquid volume	1.6 L	5.7 L	0.4 L
Stirred	No	Yes	Yes
Temperature	20-22 °C	30 °C	25-28 °C
Electron acceptor	TCE (858 μmol added/L)^1^	PCE (110 μmol added/L)^2^	TCE (278 μmol added/L)^3^
Electron donor	Methanol (4.3 mM)^1^	Butyrate (440 μM)^2^	Lactate (25 mM)^3^
Feeding frequency	14 days	2 days	4-7 days
Residence time	2 years^4^	70-80 days^5^	16-47 days^3^
Donor loading rate [(μmol/L/d)/(meeq/L/d)]	0.31/1.84	0.22/4.44	4.55/54.5
Acceptor loading rate [(mmol/L/d)/(meeq/L/d)]	0.061/0.37	0.055/0.44	0.051/0.30
Donor eeq/Acceptor eeq	5	10	180
Cobalamin amended	B_12_ (0.005 μg/L)	B_12_ (1 μg/L)	B_12_ (0.1 μg/L)
*Dhc* strain(s)	KB-1/PCE & KB-1/VC^1^	DET195^5^	ANAS (2 strains) ^3^

For electron equivalent ratios (eeq), the following eeq were utilized: PCE = 8, TCE = 6, butyrate = 18, lactate = 6, methanol = 8.

1 - [1]

2 - [29]

3 - [28]

4 - in-house data, medium exchange: 25% of volume roughly each 6 months.

5 - [20]

The availability of these metagenomes provides an opportunity to examine the microbial community composition and interactions present in these mixed microbial systems, bypassing the rigorous requirement of isolation of community members and complete genome sequencing. The rapid increase of available metagenome sequences has lead to the development of public servers and software for automated annotation and phylogenetic assignment of metagenome sequences [31-38], facilitating comparative metagenomic studies [39-45]. From metagenomic datasets, community compositions and metabolic functions can be examined. Further functional information in the form of culture-based experiments and/or transcriptomic or proteomic surveys is required to validate the predicted functions. Nevertheless, metagenomic data can suggest functional roles for organisms within a community, and inform follow-on experiments for examining these predicted roles in more detail.

Here we describe the KB-1 enrichment culture metagenome, and include a comparison with the ANAS and DonnaII enrichment consortia based on comparative metagenomics. We conduct an examination of the phylogenetic and metabolic differences and similarities among the three enrichment consortia, with a focus on the non-*Dhc* microbial population. In addition, we examine the presence and phylogenetic distribution of specific metabolic pathways of interest: cobalamin synthesis, methionine synthesis, oxygen utilization and scavenging, hydrogen production, and metabolism of the electron donors consumed by the enrichment cultures. This in-depth analysis of target pathways identifies taxonomic groups encoding synthesis pathways of required metabolites for *Dhc*, and strengthens the argument for maintenance of microbial diversity to safeguard functional redundancy within consortia.

## Results and discussion

### The KB-1 metagenome sequence

A metagenome sequence from the KB-1 *Dhc*-containing dechlorinating microbial consortium was generated by the Joint Genome Institute (JGI). The final KB-1 metagenome consists of 103 Mb of Sanger sequence data. Following assembly by the JGI’s in-house "pga.lucy" assembler, the unique sequence data is evenly split between assembled contigs (52.6% of sequence) and singletons (47.4% of sequence) (Table 2). The average contig size is quite short (2,356 bp), but 173 contigs have lengths greater than 10,000 bp, and 5 contigs have lengths greater than 100,000 bp. This uneven distribution of contig lengths is due to the varied levels of enrichment of the organisms within the KB-1 culture, whereby a dominant organism (e.g., *Dehalococcoides*) is over-represented in the community DNA and hence the metagenome sequence, yielding deeper coverage and assembly of longer contigs.

**Table 2:** General features of the metagenome datasets

**Feature**	**KB-1**	**DonnaII**	**ANAS**
Type of sequencing	Sanger	454	454 & Sanger
Total number of bases pre-assembly	106,515,530	930,446,714	330,964,688
Number of contigs	6,361	47,030	10,807
Total length of contigs (bp)	14,988,108	24,573,718	30,615,713
Number of singletons	18,629	105,608	15,486
Total length of singletons (bp)	13,487,233	57,708,799	10,450,264
Largest contig (bp)	155,970	121,460	921,258
Average contig size (bp)	2,356	522	2,832
Average G + C content (%)	52.33	52.28	51.91
Protein coding genes	40,766	194,527	60,992
- with COGs	21,857	116,001	39,920
- connected to KEGG pathways	8,077	36,685	11,878
rRNA genes (5 S/16 S/23 S)	18 (7/5/6)	185 (11/62/112)	40 (23/8/9)
tRNA genes	330	818	525
CRISPR count	48	7	57
**MG-RAST data**			
% *Dhc* in culture*	43.7	31.3	18.2
Metagenome size (bp)*	106,508,248	916,191,214	330,396,345
Average read length*	958	477	547
Number of sequences*	111,162	1,920,396	603,841
Number (%) identified for metabolic analysis^†^	63,352 (57.0)	363,424 (18.9)	222,012 (36.8)
Number (%) identified for phylogenetic analysis^†^	88,888 (80.0)	540,785 (28.2)	294,470 (48.8)

* = post-MG-RAST preprocessing, which removed duplicate reads and nonsense reads from the datasets.

† = maximum e-value of 1x10^-5^, minimum alignment length ~100.

The KB-1 metagenome was sequenced from three separate clone libraries generated from DNA samples gathered at different dates. The original 3 kb-insert clone library was generated for creation of a shotgun microarray, while the subsequent two DNA samples were gathered specifically for metagenomic sequencing by the JGI, for 3 kb and 35 kb libraries. As a result, the expected community dynamic of the metagenome is a blend of the three DNA samples at the proportions to which they contributed to the final sequence dataset (Figure 1A).

A degenerate primer-based clone library of reductive dehalogenase genes generated previously [27] identified 14 unique reductive dehalogenase homologous genes in the KB-1 consortium, named KB1_rdhA1 through A14. Sequencing of the KB-1 metagenome resulted in the identification of a further 18 complete reductive dehalogenase homologous sequences, and 3 partial *rdh* sequences, bringing the total number of identified reductive dehalogenases in the KB-1 consortium to 35 (Additional file 1: Table S1).

In addition to acting as a general blueprint for the community, the KB-1 metagenome sequence has been instrumental in informing several targeted studies [46,47].

### Comparative metagenomics of KB-1 with ANAS and DonnaII

The role of the non-dechlorinating community in *Dhc-*containing enrichment cultures is primarily to ferment the electron donor to H_2_. The additional processes contributing to the more robust dechlorination in mixed cultures compared to *Dhc* isolates are not well defined. In order to provide context to an examination of the roles of the KB-1 enrichment culture’s supporting community, we conducted a comparative metagenomic analysis of the three available dechlorinating enrichment culture metagenome sequences. The Joint Genome Institute conducted all three metagenome sequencing projects, under different modes of sequencing (see Table 2 for a comparison of metagenome properties).

The assembled datasets and the complete set of raw reads from the three metagenomes were uploaded as separate datasets into the MG-RAST server [33] for automated annotation and phylogenetic assignment. The distribution of different taxonomic groups among the three metagenomes as annotated by MG-RAST is depicted in Figure 1B. SEED data on the KB-1 consortium community composition was in general agreement with qPCR and clone library data (Figure 1A, [30]) with multiple rare organisms (>1% of the sequence) in the SEED classifications compared to the clone library. We used a hybrid taxonomic classification algorithm, RITA [48], to provide an independent assessment of the phylogenetic affiliations of the assembled datasets. RITA considers evidence from homology (via nucleotide vs. nucleotide and translated nucleotide vs. protein) and composition [49], and generated classifications that correlate well with the MG-RAST analysis on the raw read data (Figure 1B, Pearson correlation coefficient = 0.930). The main difference between these two sets of phylogenetic assessments is the proportion of *Dhc* within the datasets, where the annotated raw reads (MG-RAST) provide a more accurate depiction of the enrichment levels of *Dhc* within these consortia. Due to the small genome size and high enrichment levels, the *Dhc* contigs have higher read depths, causing the proportion of *Dhc* sequence in the assembled data to drop. Despite the differences in datasets and classifiers, the two methods largely agree on the taxonomic affiliations of the sequence data.

The MG-RAST analysis included generation of rarefaction curves for the unassembled metagenome sequences, which indicated that none of the three metagenomes have been sequenced to saturation (see MG-RAST metagenome projects for plots and data [MG-RAST IDs 4450840.3 (KB-1 raw reads), 4451142.3 (KB-1 assembled), 4451655.3 (ANAS raw reads), 4478350.3 (ANAS assembled), 4451259.3 (DonnaII raw reads) and 4447020.3 (DonnaII assembled). The distribution and diversity of the taxa identified from the three enrichment cultures indicates that certain organisms have been sequenced at much higher depths (Figure 1B). Combined with the absence of close reference genomes for these organisms, it is difficult to estimate the amount of missing information for each organism, meaning that all subsequent analyses are unable to distinguish between missing sequence data and true gene absences within these organisms.

For all subsequent pathway analyses, higher-level taxonomic assignments (phylum or class) were used to describe the presence/absence of pathways in order to reduce the effects of database bias in taxonomic assignments by MG-RAST. It must be noted that in all cases, assignment of a taxonomic identity and putative function to a sequence is dependent entirely on the presence of a known homolog within a sequenced relative in the MG-RAST database. In light of this, identification of novel features in *Dhc* was not anticipated. Indeed, the focus of this study is on non-*Dhc* populations, and on their functional gene complements. The requirement for a homolog within the database for gene identification undoubtedly caused a higher proportion of missing information in our analyses, but was an unavoidable constraint that we have attempted to work within.

### Broad-scale phylogenetic and metabolic comparison of metagenomes

**Figure 3:**
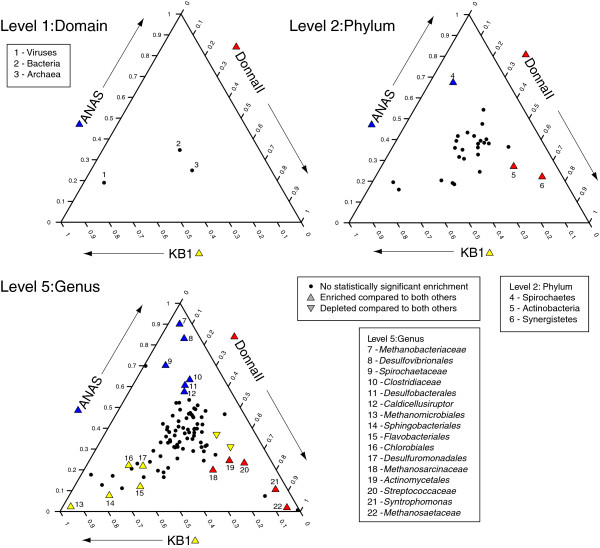
**Three-way statistical comparisons of phylogenetic differences within the enrichment culture metagenomes at different taxonomic levels.** Points are displayed as the relative proportional enrichment among the metagenomes, where the closer to a corner of the plot a point falls, the more highly proportionally enriched that category is within the metagenome affiliated with that corner (arrows). Points located in the center of the plot are not enriched in one metagenome compared to the others. Only categories in which at least one metagenome’s proportion was above a threshold of 0.1% were plotted. Taxonomic levels were taken from MG-RAST phylogenetic profiles from the SEED database. Black circles indicate categories for which no pair-wise statistical significance between proportions was determined. Coloring of points indicates statistical significance for that category given a biological effect size filter in pair-wise comparisons conducted using the STAMP interface. Colors and shapes are as follows: Yellow = KB-1, Blue = ANAS, Red = DonnaII; up-pointing triangle = enriched in that metagenome above the other two, down-pointing triangle = depleted in the metagenome colored compared to the other two.

Phylogenetic and metabolic profiles of the three metagenomes, both including and excluding reads of *Dhc* origin, were imported into STAMP [31] for pair-wise statistical comparisons (see Additional file 1: supplemental information for STAMP parameters used). The annotated metagenomes varied in size as well as proportion of unknown proteins in each dataset (35%, 24.6%, and 18.5% for DonnaII, KB-1, and ANAS respectively). For all STAMP-based comparisons, the raw read datasets were utilized, to allow quantification of gene abundances. The strengths of the combined MG-RAST/STAMP analysis are its ability to identify the potentially interesting categories deserving of closer analysis and to bypass the differences in metagenomes’ sizes and sequence type by taking dataset size into account. As an example, KB-1 exhibits a comparatively high level of enrichment in the “Virus” category (Figure 3, #1). Although the effect size is large, the total number of reads assigned to the Virus category is small (1037 hits of a total of 1769825 assigned reads for the three metagenomes combined), and the difference among the three metagenomes cannot be confidently separated from a possible sampling effect. Hence, the apparent enrichment in KB-1 is not statistically significant. On a biological note for this example, the MG-RAST database contains limited sequence information for virus genomes, which may explain the low number of hits assigned to this category.

On a taxonomic level, the STAMP analysis identifies several meaningful differences among the consortia. At the level of phylum, DonnaII exhibits a biological enrichment in Actinobacteria and Synergistetes (Figure 3, #6,7), both groups that have been detected in DonnaII [20], but not in KB-1 or ANAS [1,19]. ANAS exhibits significant enrichment in Spirochaetes (Figure 3, #4). While all three consortia contain methanogenic Archaea at similar proportions (Figure 3, #3), the taxonomic affiliations of these methanogens are quite varied. In KB-1, the dominant Archaeal group is order Methanomicrobiales (Figure 3, #13), while DonnaII contains families Methanosarcinaceae and Methanosetaceae from order Methanosarcinales (Figure 3, #18, 22) and ANAS is enriched in family Methanobacteriaceae from order Metanobacteriales (Figure 3, #7). A similar scenario is seen in the Firmicute lineages present in the consortia, with DonnaII enriched in Streptococcaceae and *Syntrophomonas* (Figure 3, #20, 21), ANAS enriched in Clostridiaceae and *Caldicellulosiruptor* (Figure 3, #10, 12) and KB-1 not enriched in any Firmicute genus.

**Figure 4:**
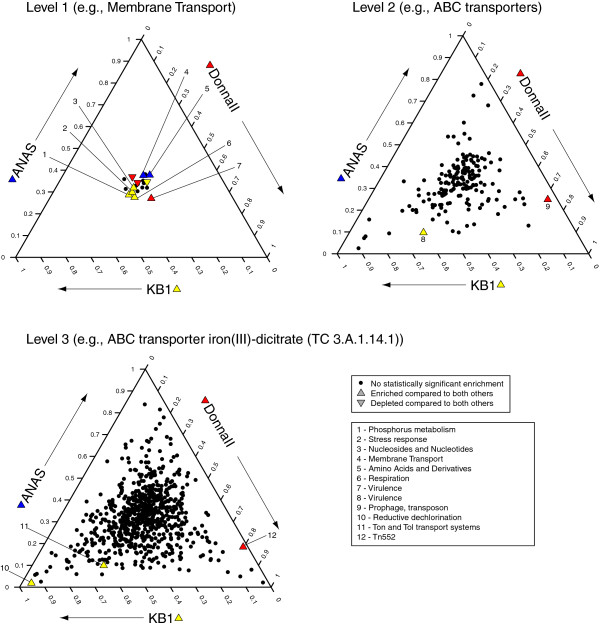
**Three-way comparisons of metabolic differences within the enrichment culture metagenomes with all *Dhc *reads removed.** Points are displayed as in Figure 3, with points here corresponding to MG-RAST assignments to categories of metabolic classification within The SEED database. The closer to a corner of the plot a point falls, the more highly proportionally enriched that category is within the metagenome affiliated with that corner (arrows). Points located in the center of the plot are not enriched in one metagenome compared to the others. Coloration and shapes are as in Figure 3. Labeled points correspond to all categories for which one metagenome was significantly enriched above the other two. For the metabolic level 1 class, the threshold for plotting was increased to 1% to reflect the lower number of possible categories.

The MG-RAST metabolic assignments for the three metagenomes with all *Dhc*-assigned reads removed were used to examine the non-*Dhc* community metabolic differences. The SEED subsystems are available at three levels of increasing specificity, where level 1 provides broad categories similar to COG designations (e.g., Membrane Transport), and levels 2 and 3 provide more specific categories (e.g., Level 2 = ABC transporters, Level 3 = ABC transporter iron(III) dicitrate (TC_3.A.1.14.1)). Several categories from the SEED level 1 exhibit differences in enrichment among the three metagenomes. However, it is clear from the triangle plot that, while these are statistically significant differences, the enrichment seen is not strong (Figure 4, level 1). A more in-depth examination was required to distinguish the nature of this significance. When examining specific subsystems (SEED level 3) there are only three categories for which one metagenome shows significant enrichment above the other two (Figure 4, level 3). DonnaII exhibits significant enrichment in the transposon 552-associated subsystem (Figure 4, #12). This SEED subsystem contains the common elements of tn552 transposons, including mobility elements (transposase *tnpA* and recombinase *binL*) as well as β-lactamase (*blaZ*) and two regulatory genes (*blaI* and *blaR*) (SEED subsystem notes). Gene-specific examination reveals this enrichment is specifically due to the Firmicute-associated β-lactamase gene (E.C. 3.5.2.6) in DonnaII. β-lactamase confers antibiotic resistance against β-lactam antibiotics, including penicillin [50]. The DonnaII bioreactor has never been exposed to antibiotics, so this resistance likely stems from the DonnaII bioreactor’s origin, the Ithaca wastewater treatment plant. Examination of the DonnaII sequences assigned to this subsystem reveals that the reads assigned to transposon-associated genes are 700x less abundant than reads assigned to the β-lactamase gene. From the assembled metagenome data, the identified β-lactamase genes are not associated with annotated transposases, either on the same contig or within the same scaffold, indicating the β-lactamase may not be associated with a transposon, and hence may not be mobile.

**Figure 5:**
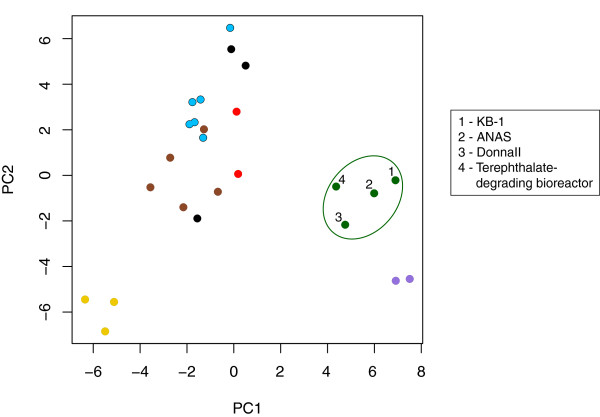
**Principal component analysis of 25 metagenomes based on frequencies of COG categories.** COG frequencies were normalized to metagenome size. Points are colored by sample type: green = contaminant-degrading microbial consortia, black = waste water/sludge samples, light blue = pristine groundwater and sediment sites, brown = soil samples, yellow = Hawaii Ocean Time Series samples, red = ammonia-oxidizing communities, purple = non-contaminant degrading microbial consortia. See Additional file 1: Table S9 for a full list of metagenomes used. All samples are publically available from the JGI IMG-M site (merced.jgi-psf.org/cgi-bin/mer/main.cgi).

The other two significantly enriched subsystems in metabolism were overrepresented in KB-1 (Figure 4, #10 & 11). The enrichment in the reductive dehalogenation subsystem in the KB-1 consortium stems from reads identified by MG-RAST as homologs to the *Geobacter* (class *∂-Proteobacteria*) reductive dehalogenases. The *Geobacter* in KB-1 is known to be capable of reductively dechlorinating PCE to *c*DCE [5]. Examination of the non-*Dhc rdh* genes detected in the three metagenomes indicated that there are no non-*Dhc rdh* genes within DonnaII, while the ANAS metagenome had some reads most similar to *Shewanella* and *Desulfitobacterium*-type *rdh* genes, indicating there may be one or more non-*Dhc* dechlorinating organisms within that culture which have not previously been detected [19,21], or that the *Dhc* strains within the ANAS consortium encode *rdh* genes that were potentially acquired through lateral gene transfer. The second subsystem enriched in the KB-1 culture is the Ton and Tol transport subsystem (Figure 4, #11). The enrichment is largely within genes in the Ton pathway for iron transport (The SEED subsystem notes, [51]), specifically within the *∂-Proteobacteria*, which for KB-1 corresponds to a *Geobacter* species. This enrichment may be linked to *Geobacter*’s requirement for corrinoids for active reductive dechlorination, as TonB has been shown to complex with the cobalamin transporter BtuB [52]. Examination of the cobalamin synthesis pathway (described below, Figure 5) suggests *Geobacter*, as the dominant *∂-Proteobacterium* in KB-1, is potentially capable of *de novo* cobalamin synthesis, but a TonB/BtuB complex may be an additional avenue for cobalamin acquisition.

In summary, the broad-scale comparison of the three metagenome sequences identified that the three enrichment consortia metagenomes differ more based on taxonomy (Figure 3, Level 5) than they do based on the proportions of the metabolic pathways encoded (Figure 4, Level 1). The identified variance in metabolic pathways was confined to either a highly specific function, as in reductive dechlorination by a non-*Dhc* organism in KB-1, or to specific resistance or mobile element markers, as in the tn552 transposon in DonnaII. Thus at a broad functional level, the communities are remarkably similar. In order to examine whether this similarity was specific to dechlorinating enrichment cultures, or merely the result of a comparison of mixed anaerobic samples, we compared the proportional distribution of genes within COG categories among 25 anaerobic mixed microbial community metagenomes using principal components analysis (Figure 5). This analysis also indicated that the three dechlorinating enrichment cultures share a higher level of similarity to each other, on a functional level, than to other, non-bioremediation-related enrichment cultures or anaerobic environmental samples.

### Examination of pathways mediating ecological interactions with Dehalococcoides

From information available from published *Dehalococcoides* genome sequences [2,7,8], a metabolic flux model of the group’s metabolism [12], and culture-based observations [18], several limitations in *Dhc* growth and metabolism have been identified. Specifically, the *Dhc* are incapable of *de novo* corrinoid synthesis, are obligately anaerobic and highly susceptible to oxygen, and are not able to transform electron donor substrates amended to mixed cultures into the H_2_ required for energy. In addition, the methionine synthesis pathway in *Dhc* has not been identified, though *Dhc* are not methionine auxotrophs [53]. We conducted in-depth examinations of selected metabolic pathways to determine how the mixed consortia may address these deficits and promote *Dhc* growth. Each pathway of interest was defined through literature searches and the SEED database, and genes pertinent to the processes mined from the MG-RAST annotated metagenomes. Annotations were kept at the phylum or class level to minimize database bias, and gene frequencies were recorded. The numeric data presented in the pathway-specific discussions is primarily from the unassembled datasets, as the assembled datasets corroborated the results from the unassembled data. Complete lists of the specific genes examined for each pathway of interest, as well as the exact gene counts for each phylum within each metagenome for both the unassembled and assembled datasets are available in the Additional file 2.

### Fermentation of electron donors to hydrogen

A significant difference among the three enrichment cultures examined is the substrate amended to the culture to serve as an electron donor (i.e., source of hydrogen) for the dechlorination reaction. In the above broad-level analysis of the metagenomes’ metabolic capacity, none of the pathways for degradation of the electron donors were proportionally enriched in a given metagenome. In a more detailed examination of the genes annotated by MG-RAST, we found that, rather than an increased proportion of the specific degradation pathway for a donor within a metagenome, there is instead a trend for increased diversity of organisms present in the culture capable of utilizing the specific donor (Table 3). We examined the presence and taxonomic attribution of the enzymes responsible for the initial steps of electron donor metabolism to identify the organisms putatively capable of the direct utilization of the three electron donors. We typically did not examine the entire pathway: once the product was a common compound in many metabolic pathways (e.g., pyruvate, acetoacetate), the organisms identified as utilizing this metabolite were not considered informative to our question.

**Table 3:** Presence of metabolic pathways for the utilization of the electron donor substrates amended to the three enrichment cultures as detected using MG-RAST annotations

	# Pathways complete/partial*			**Taxonomic Classification of Pathways**		
	**DonnaII**	**ANAS**	**KB-1**	**DonnaII**	**ANAS**	**KB-1**
**LACTATE (ANAS)**						
A - Lactate to Acetate via Pyruvate (3 genes)	2/2	1/6	0/1	α-P, Firm/Act, BC	Firm/α-P, BC, β-P, Cya, γ-P, Planc	/γ-P
B – Lactate to Ethanol (4 genes)	2/2	1/5	0/1	α-P, Firm/Act, BC	Firm/α-P, BC, β-P, Fib, γ-P	/Fib
C - L-lactate to Acetate directly (EC 1.13.12.4)	0	0	0			
**BUTYRATE (DonnaII)**						
To Acetyl-CoA and Acetoacetate (5–7 genes)	4/8	2/6	1/2	δ-P, Firm, γ-P, β-P/Act, α-P, BC, Cflx, CrenA, EurA, Fib, Fus	δ-P, Firm/Act, α-P, β-P, EurA, Fus, γ-P	Firm/BC, δ-P
**METHANOL (KB-1)**						
A - to Formaldehyde (ECs 1.1.99.8/1.11.1.6/1.11.1.7)	9	6	6	Act, BC, β-P, DT, δ-P, ϵ-P, EurA, Firm, γ-P	BC, β-P, δ-P, EurA, Firm, γ-P	BC, β-P, δ-P, EurA, Firm, γ-P
B - to Methyl-CoM, eventually to coenzyme M (4 genes)	1	1	1	EurA	EurA	EurA

Abbreviations are as follows: Act = Actinobacteria, α/β/δ/ϵ/γ-P = Proteobacteria, BC = Bacteroidetes/Chlorobi group, Cya = Cyanobacteria, Cflx = Chloroflexi, CrenA = Crenarchaeota, *Dhc* = *Dehalococcoides*, DT = *Deinococcus*/*Thermus* group, EurA = Euryarchaeota, Fib = Fibrobacteres/Acidobacteria, Firm = Firmicutes, Fus = Fusobacteria, Planc = Planctomycetes, Therm = Thermotogae.

*partial – 40% or greater of pathway must be detected, for lactate to acetate conversions, initial step was required to be present.

ANAS is amended lactate as an electron donor, which can be metabolized via pyruvate to acetyl-CoA. From acetyl-CoA, either acetate and hydrogen or ethanol can be formed (KEGG pathway: pyruvate metabolism, Additional file 2: Table S3). Another alternative is for lactate to be metabolized through lactaldehyde to propionate. The SEED database does not contain the NADH 1,2-propanediol oxidoreductase required for propionate production. BLAST-based searches of the assembled datasets for this gene indicate it is not present in any of the three metagenomes. This is reasonable, as under low hydrogen partial pressures, and in the presence of methanogens, the energetically favored pathway is via lactate dehydrogenase to pyruvate with acetate as an end product [54], which is what has been predicted for the ANAS culture [55]. ANAS contains a higher number of taxa encoding this pathway compared to the other two metagenomes (Table 3). DonnaII, by comparison, utilizes butyrate as an electron donor, which is ultimately converted to acetyl-CoA and acetoacetate and hydrogen (KEGG pathway: butanoate metabolism, Additional file 2: Table S4). The observed trend of organismal diversity exists for butyrate degradation as well, with DonnaII having more hits to distinct phyla associated with this process. KB-1 is provided methanol as an electron donor, which can be transformed to formaldehyde by acetogens or to methyl-coenzyme M en-route to methane formation by methanogens (KEGG pathway: methane metabolism, Additional file 2: Table S5). Here, the number of taxonomic groups with complete methanol degradation pathways is highest for DonnaII, with ANAS and KB-1 exhibiting the same number of groups. The electron donor for each enrichment culture was originally chosen based on comparisons of several different donors, which resulted in three different substrates being independently chosen [21,26,56]. Interestingly, this analysis predicts that each metagenome still encodes the capacity to utilize any of the electron donors examined here, despite years of exposure to only one of the three substrates. This is valuable information, as a culture’s ability to metabolize multiple electron donors increases its flexibility as a bioremediation tool, though this inferred function would need to be verified prior to implementing a change in electron donor during bioremediation.

The required electron donor for *Dhc* reductive dechlorination is H_2_, making it the central intermediate for dechlorination activity in the three consortia. Synthesis of H_2_ is primarily catalyzed by hydrogenases, a family of enzymes which can reversibly convert hydrogen cations to H_2_[57,58]. The three enrichment consortium metagenomes encode a wide diversity of hydrogenases, including nickel-only, nickel-iron, and iron-only hydrogenases, primarily identified in *Dhc*, the Firmicutes, Euryarchaeota, and the *∂-*Proteobacteria (see Additional file 1: Table S2 for a more detailed analysis of the hydrogenase family). The identified hydrogenases did not show specific taxonomic trends or enrichment in any of the three metagenomes.

### Corrinoid cofactor synthesis

*Dhc*’s inability to synthesize corrinoid cofactors *de novo* for reductive dehalogenases required for energy generation means that these cofactors must be scavenged from the environment for *Dhc* growth [2,12]. In laboratory cultures, cobalamin is typically amended to the media in the form of vitamin B12 (cyanocobalamin) (see Table 1), but in the natural environment, organisms coexisting with *Dhc* must synthesize it.

**Figure 6:**
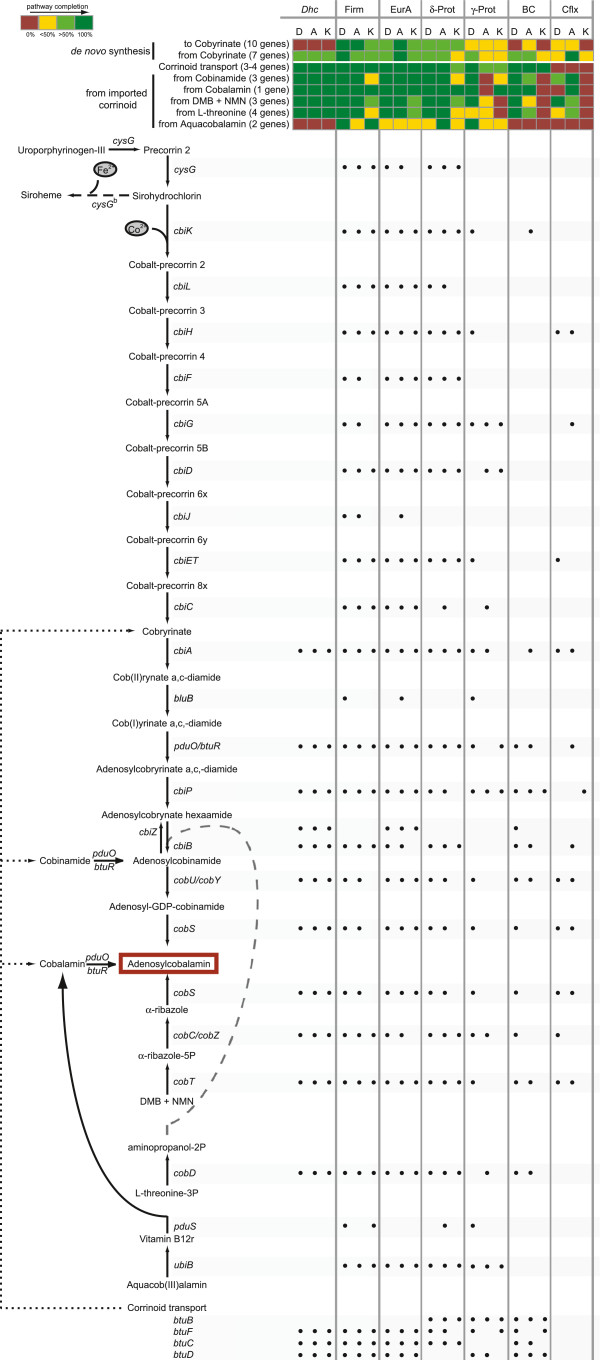
**The anaerobic cobalamin synthesis pathway with gene distributions within the three metagenomes by taxonomic classification.** The heat map depicts presence/absence of cobalamin synthesis pathways where red = genes absent; yellow = partial pathway, less than 50% of genes detected; light green = partial pathway, greater than 50% of genes detected; and dark green = complete pathway detected. Black circles indicate the specific genes detected for each microbial group. Abbreviations: DMB = 5,6-dimethylbenzimidazole, NMN = nicotinamide mononucleotide, *Dhc* = *Dehalococcoides*, Firm = Firmicutes, EurA = Euryarchaeota, δ/γ-Prot = Proteobacteria, BC = Bacteroidetes/Chlorobi group, Cflx = Chloroflexi.

An examination of the prokaryotic corrinoid synthesis pathway [59] within the three metagenomes demonstrated the absence of a complete *de novo* synthesis pathway in the strains of *Dhc* contained in these cultures. The beginning of the anaerobic corrinoid synthesis pathway, from uroporphyrinogen-III or siroheme through to cobyrinate (10 genes of a total of 17) is categorically absent from all sequenced *Dhc* genomes, including strain 195 from the DonnaII consortium [8,12]. An in-house draft genome of the *Dehalococcoides* in KB-1 also lacks homologs to this portion of the corrinoid synthesis pathway. In addition, all isolated *Dehalococcoides* strains require the addition of cobalamin for growth, including strain 195, two strains from ANAS, and an in-house isolate from KB-1 [14][13]. In contrast, the genes required for synthesis of adenosylcobalamin from partially synthesized corrin-containing products including cobyrinate, cobinamide, 5,6-dimethylbenzimidazole, and cobalamin are all present within *Dhc* in these metagenomes and sequenced genomes, as are the genes for import of these corrin-containing precursors into the cell (Figure 6, *Dhc* column). An exception to this is aquacobalamin, which does not appear to be utilized as a precursor by *Dhc*. This indicates that while *Dhc* are typically incapable of *de novo* synthesis of a corrinoid [12], they encode multiple options for import and subsequent adaptation of varied cobalamin molecules, suggesting *Dhc* are highly versatile in their ability to scavenge corrinoids from an environment and repurpose them for insertion in a reductive dehalogenase enzyme.

An examination of cobalamin synthesis in the non-dechlorinating members of the enrichment cultures yielded no evidence of the aerobic corrinoid synthesis pathway, which involves a later addition of the cobalt cation compared to the anaerobic synthesis pathway [59]. The complete *de novo* cobalamin synthesis pathway (all 17 genes) was detected in the Euryarchaeota in DonnaII and ANAS, and the Firmicutes in ANAS (Figure 6, bottom portion, see Additional file 2: Table S6 for more detail). KB-1 showed near-complete representation of the pathway for the Euryarchaeota (13 of 17 genes detected) and both DonnaII and KB-1 exhibited near-completion of the pathway in the Firmicutes (>50% of genes detected, Figure 6). The only other phylogenetic group with a significant proportion of the pathway detected was the *∂-*Proteobacteria, (14, 15, and 10 of 17 genes detected for DonnaII, ANAS, and KB-1 respectively, Figure 6). The incomplete detection of this pathway in the *∂-*Proteobacteria is likely the result of sampling effects, as the *∂-*Proteobacteria represent a smaller proportion of each of the metagenomes compared to the Firmicutes and Euryarchaeota. Further arguments in favour of a complete corrinoid synthesis pathway within the *∂-*Proteobacteria in these enrichment consortia include the uneven distribution of the “absent” genes, which argues against a possible hand-off of a partial product for completion in a different organism; there is no specific break point in the detected pathway in the *∂-*Proteobacteria as there is for *Dehalococcoides*. In addition, in contrast to sequenced *Dehalococcoides* genomes, which do not encode the upper portion of the corrinoid synthesis pathway, typically complete genomes of *∂-*Proteobacteria (e.g., the Geobacteraceae) do encode a complete cobalamin synthesis pathway [60,61]. It is possible the pathway is indeed incomplete within the *∂-*Proteobacteria present in these enrichment cultures, but the available evidence does not favour this hypothesis. The heatmap in Figure 6 depicts the extent of the corrinoid synthesis pathway in non-dechlorinating members, either *de novo* or from various precursor products. Aside from synthesis, import and conversion of corrin-containing molecules functional genes are also primarily found in the Euryarchaeota, the Firmicutes, and the *∂-*Proteobacteria.

### Methionine synthesis

Despite published experimental evidence for methionine synthesis in *Dhc*[53], no known methionine synthesis or uptake genes have been identified in *Dhc* genomes [2,12]. *Dhc* are capable of methionine uptake from the environment [62], and hence may take advantage of exogenous methionine produced by non-dechlorinating organisms. The organisms present in the enrichment cultures predicted to contain the various methionine synthesis pathways are summarized in Table 4 (see Additional file 2: Table S7 for more detail). In general, methionine synthesis is predominantly encoded by the Firmicutes in all three metagenomes, despite the enrichment cultures containing different Firmicute genera.

**Table 4:** Presence of methionine biosynthesis pathways in the three enrichment cultures as detected using MG-RAST annotations

	# Pathways complete/partial*			**Taxonomic Classification of Pathways**		
**Pathway**	**DonnaII**	**ANAS**	**KB-1**	**DonnaII**	**ANAS**	**KB-1**
**Methionine synthesis**						
Methylation pathway (from methylene-tetrahydrofolate and L-homocysteine)	2	3	3	Firm, γ-P	δ-P, Firm, γ-P	BC, δ-P, Firm
betaine-homocysteine S-methyltransferase (BhmT)	0	0	0			
**Uptake of Methionine**						
Methionine transporter MetT	5	3	2	δ-P, Firm, Fuso, γ-P, Spiro	δ-P, Firm, Fuso	BC, γ-P
ABC Met transporter (3 components)	5/2	4/2	0/1	α-P, β-P, DT, ϵ-P, Firm/Act, γ-P	α-P, ϵ-P, Firm, γ-P/Act, β-P	None/Firm

A full diagram of this system is available through the SEED viewer at ( http://seed-viewer.theseed.org/seedviewer.cgi). Taxonomic abbreviations are as in Table 3.

*partial – 40% or greater of pathway must be detected.

Based on genome and metagenome sequence data, the enzymes *Dhc* are utilizing to generate or acquire methionine are divergent from any currently known methionine synthesis enzymes. Identification of the *Dhc* genes active in methionine synthesis and transport will require targeted experiments examining transcriptomics or protein expression profiles under growth conditions lacking methionine and/or in co-cultures with the Firmicute genera highlighted here as putative methionine producers.

### Oxygen tolerance and scavenging

The maintenance of an anaerobic environment is critical for continued growth of *Dhc*, of which all known strains are obligate anaerobes. The presence of oxygen leads to a complete loss of reductive dechlorination activity, which is typically irreversible in pure cultures [14,18]. Based on the SEED annotations for the three metagenomes, the *Dhc* strains present contain two mechanisms for scavenging oxygen free-radicals: an [Mn]-superoxide dismutase (SOD) and the ruberythrin/rubredoxin putative scavenging system (Table 5, Additional file 2: Table S8). The *Dhc* sequenced genomes do not encode any identified mechanism for the direct utilization of oxygen.

**Table 5:** Presence of oxygen-scavenging mechanisms within the three enrichment cultures

	**Presence**			**Taxonomic Classification**		
	**D**	**A**	**K**	**DonnaII**	**ANAS**	**KB-1**
**Direct O**_**2**_**removal**						
Glycolate oxidase	-	+	-		Cya	
Cytochrome c oxidase	+	+	+	Act, β-P*, Cya*, δ-P*, ϵ-P*, Firm*	γ-P*	δ-P, ϵ-P*
Cytochrome d ubiquinol oxidase	+	+	+	Act*, BC, δ-P*, ϵ-P*, Firm	BC, δ-P	δ-P, Firm
Ferroxidase	+	-	-	Firm		
**Radical O**_**2**_**species**						
Catalase	+	+	+	Act, BC, β-P, DT, δ -P, ϵ-P, EurA, Firm, γ-P	BC, β-P, δ-P, EurA, Firm, γ-P	BC, β-P, δ-P, Eury, Firm, γ-P
Peroxidase	+	+	+	β-P, δ-P	δ-P, Firm, γ-P	γ-P
Cytochrome c551 peroxidase	+	+	-	ϵ-P, γ-P	ϵ-P, γ-P	
Glutathione peroxidase	+	+	+	α-P, Firm	Firm	Cya, Firm, Spiro
[Cu-Zn]-SOD	+	+	-	BC, Firm,	α-P	
[Mn]-SOD	**+**	**+**	**+**	α-P, β-P, Cya, DT, ***Dhc***, Firm, γ-P, Therm	***Dhc***, Firm, γ-P, Therm	***Dhc***, EurA, γ-P
[Fe]-SOD	+	+	+	α-P, BC, β-P, Cya, δ-P, γ-P	β-P	β-P, δ-P
[Mn/Fe]-SOD	+	+	-	EurA	EurA	
Superoxide reductase	+	+	+	δ-P, EurA, Firm, Therm	δ-P, EurA, Firm	δ-P, Firm
**Putative scavenging systems**						
Ruberythrin	**+**	**+**	**+**	BC, ***Dhc***, δ-P, ϵ-P, EurA, Firm	BC, ***Dhc***, δ-P, EurA, Firm	BC, ***Dhc***, δ-P, EurA, Firm
Rubredoxin	**+**	**+**	**+**	***Dhc***, ϵ-P, EurA, Firm, γ-P	***Dhc***, δ-P. EurA, Firm	***Dhc***, EurA
Rubredoxin-oxygen oxidoreductase	+	+	+	δ-P, EurA, Firm	δ-P, EurA, Firm	δ-P, EurA, Firm

Taxonomic abbreviations are as in Table 3.

* = partial representation of a multi-subunit enzyme complex from MG-RAST annotation.

The ability of the mixed cultures to mitigate oxygen free radical damage is significantly more robust than that of isolate *Dhc* strains *in vivo*. In support of this, each metagenome had evidence for at least 2 oxidases, a catalase, peroxidase, multiple kinds of SODs, superoxide reductase, and multiple ruberythrin/rubredoxin scavenging systems in multiple phylogenetic groups (Table 5, Additional file 2: Table S8). The main organisms encoding these oxygen scavenging systems are the *∂-*Proteobacteria, the Firmicutes, and the Euryarchaeota, with Actinobacteria contributing in DonnaII as well. From this, it seems reasonable to conclude that mixed enrichment cultures are more robust to exposure to oxygen because they encode many more enzymes for the complete removal of oxygen and damaging free radical species. Even if only a fraction of these genes are expressed as active proteins, it would still represent a substantial increase in oxygen scavenging mechanisms at work compared to pure *Dhc* isolate cultures.

## Conclusions

Examination of the metabolic and phylogenetic differences among three dechlorinating enrichment cultures revealed that, despite substantial statistically significant differences in phylogenetic groups present, the enrichment cultures show a highly conserved relative abundance of different metabolic pathways. The statistically significant metabolic differences among the three cultures’ non-*Dhc* populations were restricted to small differences in genes encoding reductive dechlorination and, as seen in many comparative metagenomic studies, the mobile element-associated genes [42,44,63]. In addition, a comparison of the metabolic signatures in the metagenomes of a wide variety of anaerobic microbial consortia confirmed that this conserved metabolic profile in the contaminant-degrading consortia is distinct from those found in non-contaminant-degrading anaerobic microbial communities (Figure 5).

In particular, the metabolic functions important in supporting *Dhc* growth were primarily encoded by the Firmicutes, the Euryarchaeota, and the *∂-*Proteobacteria, with different genera of each enriched in the three consortia (see Figure 2 for an overview of all of the systems examined here). Our results corroborate earlier enrichment culture comparison hypotheses that a diversity of genera can inhabit overlapping functional niches [25,64].

We postulate that these taxonomic groups are highlighted in the examined pathways because cultures containing them have been best able to support *Dhc* growth; laboratory enrichment for *Dhc* activity over time has required the parallel enrichment of these organisms. This analysis identifies a new importance for the Euryarchaeota in these enrichment cultures: while the Euryarchaeota are never the sole predicted source of a metabolite, it is clear from these examinations that they encode pathways predicted to provide essential nutrients, including corrinoids, to the *Dhc.* The euryarchaeotal contributions to *Dhc* growth may mitigate or even outweigh their role as H_2_ competitors with the *Dhc*, particularly when excess electron donor is present, allowing them to remain abundant in mixed cultures without adverse effects on *Dhc* growth and dechlorination. The pathway-specific examinations demonstrated that the primary communities maintained in these enrichment consortia metabolize methanol, lactate, or butyrate such that a niche for *Dhc* growth is created. Notably, each enrichment consortium is maintained on an electron donor chosen for culture performance [21,26,56], which has seemingly maintained increased organismal diversity related to donor substrate metabolism in at least two of the three cultures.

In all of the above examinations, it must be reiterated that the identified functions and phylogenetic assignments are predicated on the presence of homologs within the database utilized, and represent predicted functions only. The absence of specific genes on organisms’ genomes cannot be separated from missing data given the unsaturated status of the metagenome sequences. Lateral gene transfers will occlude correct taxonomic identifications. Given the lack of sequence saturation, it is also possible further sequencing of these communities could alter the enrichments identified here. As this analysis relies solely on genomic material, the active function of the pathways examined cannot be confirmed without further proteomic or expression data. Instead, what we have presented here represents a preliminary identification of potential organisms, genes, and pathways of interest within dechlorinating microbial consortia that may serve as guidance for subsequent targeted studies.

Each of these enrichment consortia represents a robust ecosystem allowing an examination of the global halogen cycle, where dechlorination is maintained by the collective activities of the microbial community. The metabolic differences between the enrichment consortia are subtle, which was unexpected given the consortia originated from three disparate environments and have been maintained under different conditions for many years. Taken together, the observations described here illustrate the importance of functional redundancy within dechlorinating enrichment cultures.

## Methods

### Culture and metagenome information

The KB-1 consortium was maintained in batch culture in defined mineral medium [65] with trichloroethene (TCE) as electron acceptor and methanol as electron donor. The culture was routinely allowed to dechlorinate TCE completely to ethene prior to a new amendment of acceptor/donor approximately every two weeks. The DonnaII reactor and ANAS semi-batch reactor were maintained as described previously [19,20]. See Table 1 for a summary comparison of maintenance conditions, and Table 2 for metagenome sizes and modes of sequencing used.

For the KB-1 metagenome, DNA was extracted from the T3 MP1 KB-1 culture just after completion of a dechlorination cycle using a Cetyl trimethylammonium bromide (CTAB) protocol [66] with volumes scaled up for higher yield as described in the alternate protocol, omitting subsequent cesium chloride gradient centrifugation steps. Clone libraries with 35-kb and 3-kb inserts were created by the JGI using their in-house protocols ( http://www.jgi.doe.gov/sequencing/protocols), and an additional 3 kb short insert library generated for construction of a shotgun metagenome microarray was constructed by Genome Atlantic. A total of 103 MB of metagenome sequence was generated on AB13730xl Sanger sequencers from the three clone libraries. The metagenome was assembled using the JGI’s in-house bacterial assembly pipeline, utilizing lucy [67] for vector and quality screening. The KB-1 metagenome sequence and assembly were made publically available by the JGI on May 2^nd^, 2009 ( http://genome.jgi-psf.org/aqukb/aqukb.download.ftp.html). Community composition of the three KB-1 DNA samples utilized for sequencing the KB-1 metagenome was determined by Dr. Alison S. Waller using qPCR [30].

The DonnaII metagenome was generated from 454 Titanium libraries according to the JGI’s in-house protocols ( http://www.jgi.doe.gov/sequencing/protocols). Metagenome assembly was conducted using the Newbler program from Roche [68]. The sequence data, including an in-house assembly draft for the DonnaII metagenome was made publically available by the JGI on January 31^st^, 2012 (IMG-M taxon ID: 2032320001 ( http://img.jgi.doe.gov/cgi-bin/m/main.cgi)).

The ANAS metagenome was composed of a combination of Sanger sequencing and Titanium 454 sequencing as described above. The sequence data, including an in-house assembly draft for the ANAS metagenome was made publically available by the JGI on August 20^th^, 2009 (IMG-M taxon ID: 2014730001 ( http://img.jgi.doe.gov/cgi-bin/m/main.cgi)).

### Comparative metagenome analysis

The raw reads from all three metagenomes as well as the assembled datasets were submitted to the MG-RAST server [33] for automated annotation utilizing the SEED database [34]. All phylogenetic and metabolic profiles discussed herein were generated utilizing a maximum e-value of 1x10^-5^ and a minimum alignment length match of 100 required, criteria designed to reduce noise and poorly supported assignments, but to still allow imperfect matches between sequences and novel homologs. A global criterion is not ideal for preventing any erroneous annotations, but is required when working with datasets of this size. Vector sequences were identified and removed from the analysis (see Additional file 1: materials and methods). In order to facilitate certain comparisons of the non-*Dhc* communities, all reads assigned to *Dhc* under MG-RAST’s phylogenetic profiles for the SEED, Silva LSU, Silva SSU, RDP, and Greengenes databases were removed from the datasets, and a second automated annotation conducted for the “*Dhc*-subtracted” metagenomes.

The assembled datasets were input to the RITA pipeline [48] to generate a second assessment of community composition. RITA combines homology-based predictions with the Naïve Bayes approach to compositional classification used in [49] to generate predictions with increased precision relative to a one-step BLASTN search performed by MG-RAST. The RITA pipeline was used to assign taxonomy to fragments using a reference database of 1479 genomes using the following matching protocols: (i) agreement between the best match with UBLASTX (maximum e-value = 10^-15^) [69] and the Naïve Bayes classification of a fragment; (ii) a difference of at least ten orders of magnitude between the e-value of the best match and that of the second-best matching group; (iii)-(iv) same as (i)-(ii), but using BLASTN instead of UBLASTX; and (v) matches based on the Naïve Bayes prediction alone. Fragments assigned to set (v) have a much lower confidence than sets (i)-(iv) which are based on homology.

Three-way comparisons were conducted among the metagenomes, adapting the method described by Tringe *et al.*[45] for MG-RAST output files. In brief, subsystem counts (for metabolic analyses) or taxonomic counts (for a chosen phylogenetic class analysis) were converted to a proportional count for each metagenome. Pseudocounts proportional to the size of each dataset were added to prevent selection for rare categories [45]. For each category, the relative proportions for the three metagenomes were normalized to one. The data were displayed as a barycentric plot, allowing 3-dimensional data to be displayed in 2 dimensions utilizing scripts developed by Tringe *et al.*[45]. Trends seen in this three-way comparison test were confirmed by the pair-wise statistical testing in STAMP with a p-value of 0.05 (full statistical methods available in the Additional file 1: methods), allowing the biological effect size filtering to be indirectly applied to observed three-way differences.

### Specific pathway comparisons

For each of the identified pathways or functions of interest, a literature search was conducted to determine all enzymes associated with the system. The SEED database was searched for the subsystem location(s) of the identified genes using enzyme commission (EC) numbers where available, and enzyme names or synonyms as listed in BRENDA ( http://www.brenda-enzymes.org) where EC numbers were not available or omitted in the SEED. The complete tabular data for each of subsystems on the resultant list were exported from MG-RAST for each of the three metagenomes.

The SEED phylogenetic identifiers associated with each sequence read were converted from the SEED-specific nomenclature to the NCBI taxonomic identifier, and a phylum-level taxonomic assignment was added. *Dhc* was designated as a phylum-level classification to distinguish between reads assigned to *Dhc* and reads assigned to other *Chloroflexi*. The presence and proportion of the genes of interest across phylogenetic groups were examined (summary tables available in Additional file 2).

## Abbreviations

PCE: Tetrachloroethene; TCE: Trichloroethene; DCE: Dichloroethene; VC: Vinyl chloride; SOD: Superoxide dismutase.

## Competing interests

The authors declare they have no competing interests.

## Authors’ contributions

LAH conceived of the experiments, carried out the analyses, and drafted the manuscript. RGB and ARR assisted with the analyses and helped to draft the manuscript. RER and EAE participated in the design of the experiments, and helped to draft the manuscript. All authors read and approved the final manuscript.

## Supplementary Material

Additional file 1 **Contains supplemental methods as referenced in the main text, and a discussion of the hydrogenase distribution within the enrichment consortia, including supplemental tables S1, S2, and S9 **[58,70]. Click here for file

Additional file 2 **Contains supplemental tables S3-S8; detailed tables of the pathways discussed in the manuscript, including all genes implicated and the number of assigned reads to each gene from each phylum examined.** Presented as a single MS Excel workbook, with each sheet corresponding to a different pathway of interest. Each enzyme examined within a pathway is listed in the left-most column, with number of assigned reads from MG-RAST for each phylum within each metagenome listed. Metagenomes are indicated as follows; D = DonnaII, A = ANAS, K = KB-1. Enzymes found within the metagenomes are highlighted in green, while enzymes absent from specific phyla are highlighted in red. Enzymes of interest may be absent either because they are not present in the SEED database (denoted with (NOT FOUND) in the enzyme name), or were not detected in the metagenome datasets. Click here for file

## Author details

^1^Department of Cell and Systems Biology, University of Toronto, Toronto, Canada. ^2^Faculty of Computer Science, Dalhousie University, Halifax, NS, Canada. ^3^Civil & Environmental Engineering, Cornell University, Ithaca, NY, USA. ^4^Department of Chemical Engineering and Applied Chemistry, University of Toronto, Toronto, ON, Canada.
