# Supplementary material for: Comparative metagenomics of three Dehalococcoides-containing enrichment cultures: the role of the non-dechlorinating community
Source: BMC Genomics. 2012 Jul 23;13:327. doi: 10.1186/1471-2164-13-327 (PMC3475024; doi:10.1186/1471-2164-13-327)
Supplement: Additional file 1 — Contains supplemental methods as referenced in the main text, and a discussion of the hydrogenase distribution within the enrichment consortia, including supplemental tables S1, S2, and S9 [58,70]. [file 1471-2164-13-327-S1.doc]

**SUPPLEMENTAL INFORMATION (methods, results, and tables S1, S2, and S9)**

***Supplemental Methods***

*STAMP parameters for statistical analysis*

All STAMP comparisons were conducted implementing the authors’ recommended statistical rigor: significance was determined using a two-sided Fisher’s exact test with a Bonferroni multiple test correction. Confidence intervals were determined under the DP Newcombe-Wilsome method with a 0.95 confidence level set. Biological meaning was assessed with filters set to q-values of 0.05. The effect size filter 1 (Difference between proportions) was 0.5% and the effect size filter 2 (Ratio of proportions) was set to 2. For the taxonomic examination, MG-RAST output tables were altered to make “Level 5” consistent with Genera, and to upgrade the divisions of the Proteobacteria to “Level 2”, which was subsequently used as the level of phylum. *Dehalococcoides* entries were also upgraded to the level of phylum to allow discrimination between *Dehalococcoides* and other Chloroflexi bacteria.

*KB-1 18S rRNA examination*

KB-1 originally appeared to be statistically enriched in the *de novo* purine biosynthesis SEED subsystem, as well as the Domain Eukaryota. This was the result of cloning vector sequences being misannotated as Phosphoribosylaminoimidazole carboxylase ATPase (EC 4.1.1.21) from Metazoa, and *Saccharomyces* specifically.

BLASTn [70] against the NCBI 'nr' database of non-redundant nucleotide sequences confirmed the KB-1 reads identified as Phosphoribosylaminoimidazole carboxylase ATPase (EC 4.1.1.21) have a best match to known cloning vector sequence.

An 18S rDNA PCR was conducted using the universal primer 515F (GTGCCAGCAGCCGCGGTAA) and the Eukaryotic-specific primer 1209R (GGGCATCACAGACCTG). Reactions contained 0.25 mM dNTPs, 0.5 mM of each primer, 5 units of Taq polymerase (NEB), and 1x reaction buffer (NEB) in a final volume of 100 µL. A positive control sample of genomic DNA from *Arabidopsis thaliana* and a DNA-free negative control were assayed concurrent with two KB-1 DNA samples: archived DNA from the KB-1 metagenome sequencing and a more recent genomic DNA extraction.

Reaction conditions were as follows: 95 C for 1 minute, followed by 40 cycles of 94 C for 30 seconds, 55 C for 30 seconds, and 72 C for 1 minute, with a final extension of 72 C for 10 minutes.

Reaction products were examined on a 1% agarose gel. The KB-1 samples showed amplification with two distinct bands of ~600 bp and ~950 bp. The positive control showed clear amplification of one ~850 bp band. The negative control showed no amplification.

The two bands from the archived KB-1 DNA sample were gel cleaned using the Wizard SV gel and PCR clean-up system (Promega), and cloned in separate reactions using the TOPO TA vector and TOP10 chemically competent cells (Invitrogen). Clones were picked and grown overnight in LB+kanomycin (50 mg/L) media, and plasmids extracted using the GenElute HP Plasmid purification system (Sigma).

A set of 5 clones from each cloning reaction (amplified DNA band) were sent for sequencing using the vector primers T7F and M13R. Sequences confirmed the observed PCR products were the result of non-specific amplification. The larger band corresponded to sequence from a thiamine pyrophosphate (TPP) binding domain protein in *Geobacter*, while the smaller band’s clone sequences were identified as bacterial N-acetyltransferases and N-acetylneuraminate synthases.

***Supplemental Results and Discussion***

*Examination of specific metabolic pathways of interest*

*Hydrogen production*

The hydrogenases present in the three enrichment culture metagenomes were primarily identified in *Dehalococcoides*, the Firmicutes, Euryarchaeota, and the ∂-Proteobacteria (Table S2). Based on the diversity of hydrogenases in *Dehalococcoides*, it is possible that they are capable of converting pyruvate to H_2_ directly, providing an alternate source of hydrogen in the absence of external H_2_. If true, *Dhc* may only require other community members to catalyze the initial breakdown of the amended electron donor to pyruvate for hydrogenase generation of H_2_ [57]. This potential avenue for hydrogen generation would need to be confirmed through *in vivo* experimentation with a pure strain of *Dhc*, but might prove an interesting avenue for exploration. Further characterization of the hydrogenases present in the three enrichment consortia metagenomes are presented in Table S2.

Table S1: Current identified reductive dehalogenase homologous genes within the KB-1 consortium, with various gene identifiers including common names, in house identifiers from the draft genome gene IDs, and JGI locus tags. Genes homologous to RDases with known functions are noted.

|  | Name | JGI gene ID | JGI locus tag | NCBI Accession | Notes |
| --- | --- | --- | --- | --- | --- |
| 1 | KB1_1_rdhA | none | none | DQ177506 | 99% ID to C3109_4 |
| 2 | KB1_2_rdhA | 2013897470 | DCKB1_110450 | DQ177507 |  |
| 3 | KB1_3_rdhA | 2013887593 | DCKB1_11560 | DQ177508 |  |
| 4 | KB1_4_rdhA | 2013897443 | DCKB1_110180 | DQ177509 |  |
| 5 | KB1_5_rdhA | 2013897436 | DCKB1_110110 | DQ177510 |  |
| 6 | KB1_6_rdhA | none | none | DQ177511 | *bvcA* homolog*,* partial sequence in metagenome |
| 7 | KB1_7_rdhA | none | none | DQ177512 | 99% ID to C3241_7 |
| 8 | KB1_8_rdhA | 2013887591 | DCKB1_11540 | DQ177513 |  |
| 9 | KB1_9_rdhA | 2013897446 | DCKB1_110210 | DQ177514 |  |
| 10 | KB1_10_rdhA | 2013897473 | DCKB1_110480 | DQ177515 | 99% ID to C3109_9 |
| 11 | KB1_11A | none | none | DQ177516 | 99% ID to C3240_1 |
| 12 | KB1_12_rdhA | 2013897488 | DCKB1_110630 | DQ177517 |  |
| 13 | KB1_13_rdhA | 2013897479 | DCKB1_110540 | DQ177518 | *pceA* homolog |
| 14 | KB1_14_rdhA | 2013896112 | DCKB1_96900 | DQ177519 | *vcrA* homolog |
| 15 | KB1_22_rdhA | none | none |  | 99% ID to C3109_7 |
| 16 | C3241_1 | 2013897921 | DCKB1_115000 |  |  |
| 17 | C3241_2 | 2013897936 | DCKB1_115150 |  | *tceA* homolog |
| 18 | C3241_3 | 2013897942 | DCKB1_115210 |  |  |
| 19 | C3241_4 | 2013897923 | DCKB1_115020 |  |  |
| 20 | C3241_5 | 2013897930 | DCKB1_115090 |  |  |
| 21 | C3241_6 | 2013897912 | DCKB1_114910 |  |  |
| 22 | C3241_8 | 2013897907 | DCKB1_114860 |  |  |
| 23 | C3107_1 | 2013897169 | DCKB1_107470 |  |  |
| 24 | C3107_2 | 2013897174 | DCKB1_107520 |  |  |
| 25 | C3109_7 | 2013897485 | DCKB1_110600 |  |  |
| 26 | C1024_1 | 2013890159 | DCKB1_37290 |  |  |
| 27 | C3108_1 | 2013897212 | DCKB1_107910 |  | conserved, syntenic *rdh* in *Dhc* genomes |
| 28 | C338_1 | 2013887924 | DCKB1_14890 |  |  |
| 29 | C3109_4 | 2013897452 | DCKB1_110270 |  |  |
| 30 | C3109_9 | 2013897473 | DCKB1_110480 |  |  |
| 31 | C3241_7 | 2013897904 | DCKB1_114830 |  |  |
| 32 | KB1_group26 | 2013887811 | DCKB1_13760 |  |  |
| 33 | KB1_F43012 | 2013916109 | DCKB1_297220 |  | partial (721 bp) |
| 34 | KB1_F51719 | 2013918333 | DCKB1_319470 |  | partial (739 bp) |
| 35 | C3240_1 | 2013897900 | DCKB1_114790 |  | partial (894 bp) |

Table S2: Presence of hydrogenases and other genes associated with H_2_ generation in microbial organisms. Hydrogenases for which 50% or greater of the known subunits were detected are listed as present. Hydrogenase classifications are based on SEED categories adapted to the current metal-based nomenclature. Taxonomic abbreviations are as in table 3.

|  | **Taxonomic Classification** | | |
| --- | --- | --- | --- |
|  | **DonnaII** | **ANAS** | **KB-1** |
| **NICKEL-ONLY HYDROGENASES** |  |  |  |
| Nickel-dependent hydrogenase (2 subunits) | δ-P | δ-P | δ-P |
|  |  |  |  |
| **NICKEL-IRON HYDROGENASES** |  |  |  |
| NAD-reducing hydrogenase (EC 1.12.1.2) (7 subunits) | δ-P, γ-P, β-P | δ-P, Fib | δ-P |
| Uptake hydrogenase (EC 1.12.99.6) (2 subunits) | δ-P, BC, α-P, β-P, ε-P | δ-P, γ-P, α-P, β-P | δ-P, |
| [Ni/Fe] hydrogenase, group 1 (2 subunits) | ***Dhc***, Firm | ***Dhc*** | ***Dhc*** |
| Quinone-reactive Ni/Fe-hydrogenase (EC 1.12.5.1) (4 subunits) |  |  | Firm |
| Ni,Fe-hydrogenase III (2 subunits) | ***Dhc***, δ-P, Firm, β-P | ***Dhc***, δ-P, Firm | ***Dhc***, δ-P |
| Coenzyme F420 hydrogenase (EC 1.12.98.1) (3 subunits) | EurA | EurA | EurA |
| Energy conserving hydrogenase EchA (31 subunits) |  | EurA |  |
| Energy conserving hydrogenase EchB (17 subunits) |  | EurA |  |
| Carbon monoxide-induced hydrogenase (6 subunits) | Firm | δ-P, Firm | Firm |
|  |  |  |  |
| **IRON-ONLY HYDROGENASES** |  |  |  |
| Cytochrome-c3 hydrogenase (4 subunits) |  | EurA |  |
| Periplasmic [Fe] hydrogenase (EC 1.12.7.2) (2 subunits) | ***Dhc***, δ-P, Firm, BC, Fib, Spiro, Therm, γ-P | ***Dhc***, δ-P, Firm, BC, Fib, Spiro, Therm | ***Dhc***, δ-P, Firm, BC, Fib, Spiro, Therm |
| NADP-reducing hydrogenase (4 subunits) | Firm, EurA, Therm | EurA, Therm | EurA. Therm |
| [Fe] hydrogenase (Hym) (3 subunits) | ***Dhc*** | ***Dhc*** | ***Dhc*** |
| Fe-S-cluster-containing hydrogenase (2 subunits) | δ-P, Firm, ε-P | δ-P, EurA | δ-P |
|  |  |  |  |
| **OTHER HYDROGENASES** |  |  |  |
| Energy-conserving hydrogenase (ferredoxin) (6 subunits) | ***Dhc***, δ-P, Firm, EurA | ***Dhc***, δ-P, Firm, EurA | ***Dhc***, δ-P, Firm, EurA |
| Hydrogenase, group 4 (4 subunits) | ***Dhc*** | ***Dhc*** | (***Dhc***: 1 of 4 subunits) |
|  |  |  |  |
| **OTHER RELATED GENES** |  |  |  |
| Formate hydrogenlyase (Fdh)(6 subunits) | ***Dhc***, δ-P, EurA, α-P | δ-P, EurA, γ-P | ***Dhc***, EurA |

Table S9: List of metagenomes used in Figure 4. Metagenomes are publically available from the IMG-M metagenome sequence repository (merced.jgi-psf.org/cgi-bin/mer/main.cgi).

| IMG-M Taxon OID | Genome |
| --- | --- |
| 2000000000 | Sludge/US, Phrap Assembly |
| 2000000001 | Sludge/Australian, Phrap Assembly |
| 2032320007 | Atta texana internal waste dump (Dump bottom) |
| 2006207000 | Methylotrophic community from Lake Washington sediment Methane enrichment |
| 2006207001 | Methylotrophic community from Lake Washington sediment Methanol enrichment |
| 2006207002 | Methylotrophic community from Lake Washington sediment Methylamine enrichment |
| 2006207003 | Methylotrophic community from Lake Washington sediment Formaldehyde enrichment |
| 2006543005 | Methylotrophic community from Lake Washington sediment combined (v2) |
| 2007427000 | Oak Ridge Pristine Groundwater FRC FW301 |
| 2007915000 | Wastewater Terephthalate-degrading communities from Bioreactor |
| 2013843002 | Aquatic dechlorinating community **(KB-1)** (Sample 10166) |
| 2032320001 | PCE-dechlorinating mixed culture (PCEOT) **(DonnaII)** |
| 2014730001 | ANAS dechlorinating bioreactor (Sample 196) **(ANAS)** |
| 2032320004 | Soil microbial communities from FACE and OTC sites (Maryland Estuary ambient) |
| 2032320005 | Soil microbial communities from FACE and OTC sites (Oak Ridge ambient) |
| 2044078000 | Maize field bulk soil (Bulk soil sample from field growing corn (Zea mays)) |
| 2001200001 | Soil microbial communities from Minnesota Farm |
| 2021593004 | Soil microbial community from switchgrass rhizosphere |
| 2014642001 | Marine planktonic communities from Hawaii Ocean Times Series Station (HOT/ALOHA) (3_Below_base_of_euphotic) |
| 2014642002 | Marine planktonic communities from Hawaii Ocean Times Series Station (HOT/ALOHA) (5_Below_upper_mesopelagic) |
| 2014642004 | Marine planktonic communities from Hawaii Ocean Times Series Station (HOT/ALOHA) (4_Deep_abyss) |
| 2030936003 | Anammox bioreactor Anammoxoglobus propionicus |
| 2017108002 | Marine anaerobic ammonium oxidizing (anammox) community |
| 2049941001 | Mixed alcohol (MixAlco) bioreactor (40 degree reactor) |
| 2010388001 | Poplar biomass decaying microbial community |
